# Identification of Illness Uncertainty in Veterinary Oncology: Implications for Service

**DOI:** 10.3389/fvets.2019.00147

**Published:** 2019-06-05

**Authors:** Debbie L. Stoewen, Jason B. Coe, Clare MacMartin, Elizabeth A. Stone, Catherine E. Dewey

**Affiliations:** ^1^Department of Population Medicine, Ontario Veterinary College, University of Guelph, Guelph, ON, Canada; ^2^Department of Family Relations and Applied Nutrition, College of Social & Applied Human Sciences, University of Guelph, Guelph, ON, Canada; ^3^Department of Clinical Studies, Ontario Veterinary College, University of Guelph, Guelph, ON, Canada

**Keywords:** uncertainty, cancer, coping, adaptation, expectations, communication, veterinary, qualitative

## Abstract

Uncertainty has been identified as the central psychological feature of illness experiences, necessitating a variety of coping strategies to effectively manage it and successfully adapt. The purpose of this qualitative study was to determine the expectations of veterinary clients accessing oncology care services at a tertiary referral center for dogs with life-limiting cancer. The study consisted of 43 dog owners participating in 30 independent in-person single and dyadic interviews conducted with standardized open- and closed-ended questions from April to October 2009. Thematic analysis (supplemented with content analysis) was performed on transcripts of the interview discussions. Uncertainty was inadvertently identified as a central theme of the clients' experience. The diagnosis of a serious, life-limiting cancer and its treatment appeared to move clients into a world of uncertainty, which affected their feelings, thoughts, behaviors, attitudes, and personal expectations in relation to their dog, and their expectations of the oncology service. With uncertainty appraised mostly as a danger, clients appeared to employ multiple coping strategies to reduce uncertainty in the effort to adapt to the new reality of living with and caring for a dog with cancer. The need to manage uncertainty influenced their expectations of the service, specifically for information, ongoing relationships, 24-h access, and timely care. Our findings have implications for the delivery of specialty oncology services and for client welfare. When working with owners of dogs with life-limiting cancer, results suggest health care providers can facilitate the management of uncertainty to enhance clients' psychological well-being, thereby supporting clients' successful adaptation to the cancer experience.

## Introduction

Uncertainty has long been recognized as a central feature in illness experiences (1, 2). As understood across a number of disciplines,

“*Uncertainty is a dynamic state in which there is a perception of being unable to assign probabilities for outcomes that prompts a discomforting, uneasy sensation that may be affected (reduced or escalated) through cognitive, emotive, or behavioral reactions, or by the passage of time and changes in the perception of circumstances. The experience of uncertainty is pervasive in human existence and is mediated by feelings of confidence and control that may be highly specific (event-focused) or more global (a world view).”* (3).

Our nature as humans instructs us to suspend our thoughts about uncertainty and live in an assumptive world, finding therein the predictability, continuity, and coherence needed to experience the world as both safe and stable (4–6). We go about our daily lives presuming the world to be certain (6), sheltered by the belief that what has been true will continue to be true (7). There is an inherent need to believe that the world is an orderly and predictable place, and a place where what ought to happen does happen (4, 8). Events that rupture the assumptive world are transformative, opening wide the world of uncertainty (6). The diagnosis of a serious illness is one such event.

According to Michel's *Uncertainty in Illness Theory* (9, 10), uncertainty is forwarded as the central psychological feature of the illness experience, and its management the essential task in adaptation (11). According to Mishel (12–14) there are four forms of uncertainty in illness experiences: (1) ambiguity concerning the state of the illness; (2) unpredictability concerning the illness course and prognosis; (3) lack of information about an illness, its treatment, and the system of care; and (4) complexity, or lack of clarity, in what information exists. The uncertainties that characterize illness experiences begin at the diagnosis and often persist throughout the disease trajectory (15–17), varying in degree and form in proportion to circumstances (18–20).

Uncertainty, in and of itself, is not inherently good or bad (9). The meaning of uncertainty, and thus its significance, is assigned through its appraisal. Uncertainty may be appraised in three ways: as a danger, when perceived as a source of harm or threat; an opportunity, when perceived as a source of hope or possibility; or inconsequential, when perceived as irrelevant to well-being (9, 19). Because of the very nature of uncertainty, its appraisal is open to multiple interpretations (9). These are determined by an individual's dispositions, beliefs and experiences (21).

When appraised as a danger, coping strategies are applied to reduce it (9). These strategies may be problem-focused, with the aim to directly resolve the source of uncertainty, or emotion-focused, with the aim to manage the uncomfortable emotions associated with uncertainty (22). Alternatively, when appraised as an opportunity, coping strategies are applied to sustain or even increase it (9). Uncertainty thus is *managed* through the use of coping strategies to reduce, maintain or increase uncertainty in order to successfully adapt to the illness experience (19).

Problem-focused coping strategies include information seeking, vigilance, accessing social support, focusing on the positive, and living in the present (9, 22). Information seeking is recognized as the primary strategy individuals use to cope with uncertainty (23). It can be “deliberate, intentional, and often effortful” when the need to reduce uncertainty is high (24). Vigilance, which refers to the ongoing monitoring of the patient-*in-situ*, is similar in that uncertainty is reduced through data acquisition. Vigilance enables the early detection of (and therefore ability to respond to) changes that could suggest the presence of threat to well-being. Social support can assist with interpreting illness-related events, and likewise, through social comparison, broaden awareness about illness-related issues (9). Both the consciously employed strategies of optimism and living life in edited time units reduce the perception of uncertainty and thus its impact (6, 17, 25, 26). Optimism can also reduce feelings of vulnerability and increase the sense of personal control (27). Problem-focused strategies tend to be used in situations perceived to be manageable (9).

Emotion-focused coping strategies, on the other hand, are used to moderate the emotional distress associated with uncertainty in the effort to maintain emotional equilibrium. They include denial, avoidance, selective ignoring, minimizing, selective misinterpretation, and wishful thinking (9, 28), and are especially implemented when the distress level is high and the source of the uncertainty unchangeable (9, 22).

If unmanaged, uncertainty can become a significant source of psychological distress and reduce quality of life, and in this way, negatively impact adaptation to the illness experience (16, 29, 30). There is substantive evidence on the impact of uncertainty in specific populations, most notably those with cancer (16, 31–36), the most alarming, dreaded, and fear invoking of all diseases (37–40). Uncertainty is not a new topic in the field of cancer care (31), with the first reports published in the 1980s; (12, 16, 41) it continues to resonate with patients and their families (36). Although recent years have seen an expansion of the study of uncertainty (16), to date, according to the authors' awareness, uncertainty has not been explored in the veterinary context.

In a previously described (42, 43), interview-based qualitative research study, the purpose of which was to determine the expectations of clients accessing oncology care services at a tertiary referral center for dogs with life-limiting cancer, uncertainty was inadvertently identified as a primary theme of clients' experience. The promise within a rigorous approach to interview-based qualitative research is the opportunity to generate a deep understanding of an area through learning about the firsthand experiences, views, opinions and ideas of individuals (44). In contrast to quantitative methods, which deal with numbers, are primarily deductive, and seek to draw generalizable conclusions, qualitative methods deal with words, are inductive, and seek to describe and generate understandings about phenomena about which little is known (44). In the study of client expectations, the phenomenon of uncertainty emerged as a primary theme. The objective of the present article is to report on the theme of uncertainty, specifically clients' experience of uncertainty, how it influenced them, and how it shaped their expectations of the oncology service.

## Materials and Methods

### Study Design

Details of the study design, including study participants, interview structure, and data analysis, have been previously described (42, 43). The study protocol was reviewed and cleared by the University of Guelph Research Ethics Board. In brief, 30 interviews were conducted with pet owners seeking cancer treatment for their dogs at the OVC Health Sciences Center, University of Guelph, over a 7-month period (from April to October 2009). Participants, as single owners or co-owners, were prospectively recruited from the oncology care service clientele as a convenience sample and provided written consent to participate in the study.

Owners (*n* = 43) were designated as early-, middle-, or late-stage clients in relation to their dogs (*n* = 30) stage of treatment. Treatment programs ranged from 2 weeks to 6 months in duration. Those owners categorized as within the early stage were interviewed during one of the first two appointments for the treatment program; those in the middle stage were at the mid-point of the expected duration of the treatment program; and those in the late stage were in the last two visits of their treatment program. Ten interviews (duration, 1 h) regarding 10 dogs were undertaken for each treatment stage. Seventeen interviews were conducted with the primary caregiver individually and 13 with the primary caregiver and 1 other family member (9 husband-wife and 4 mother-daughter dyads).

At the time of the interview, the primary caregiver for each dog completed a short demographic form and a widely used questionnaire with established psychometric properties for measuring the human-animal bond (45). The LAPS provides a score that indicates the category of attachment of the owner to the pet as follows: score of 54.9 (SD, 9.2), very attached; score of 44.8 (SD, 10.3), somewhat attached; score of 32.6 (SD, 9.3), not very attached; and score of 26.2 (SD, 13.6), not at all attached (46).

### Sample Characteristics

Of the 30 primary caregivers, 22 (73%) were female and 26 (87%) were 30 to 64 years of age, and the mean LAPS score was 60.5 (SD, 6.55; range, 40 to 69), which indicated that most primary caregivers were very attached to their dogs. The mean age of the dogs was 8.7 (SD, 2.5) years. Each dog had one of various life-limiting cancers, including multicentric lymphoma, appendicular osteosarcoma, high grade mast cell tumor, and hemangiosarcoma, among others.

### Interview Structure

Semi-structured single and dyadic interviews were conducted to identify clients' expectations of the oncology care service. Interviews followed standardized open- and closed-ended questions as contained within 1 of 2 interview guides, the details of which have been previously described (42, 43). One interview guide was for use among early-stage clients, and the other interview guide was for use among middle- and late-stage clients. An intentionally curious, dispassionate, and nonpartisan interview stance was taken throughout the interviews. Interviews were collected by use of an audio recorder (47) and computer program (48), and transcribed verbatim by a professional transcriptionist and reviewed by the principal author (DLS) to ensure quality and accuracy. Inaudible, implied, or tense-adjusted words were square bracketed, and the names of clients, patients, and oncology service providers were replaced with curly bracketed non-identifying descriptors for the purposes of data reporting.

### Data Analysis

Thematic analysis (49), was performed group wise by the stage of the treatment to identify, analyze, and report the primary patterns or themes across the data. To start, interesting features of the data were coded to generate a listing of codes supported by data extracts. The codes were then sorted into subthemes, resulting in a collation of the relevant data extracts within each subtheme. Related subthemes were drawn together via mapping to create main themes. The interrelationships between these themes were then defined and described. As thematic analysis is a recursive rather than linear process, involving back and forth movement between the phases of analysis (49), this process required multiple passes through each transcript, between transcripts, and between the 3 stages of transcripts, looking for similarities and differences. The coding was consistently performed by the primary author (DLS) and reviewed periodically with comparison coding with co-authors. Likewise, the process of theme generation and mapping was reviewed with the coauthors.

With “uncertainty” identified as a primary theme, content analysis, a procedure to quantify qualitative data (conversation) through the examination of frequency counts of words or concepts (50) was performed to determine the relative extent of uncertainty within the three stages of treatment. Across the dataset, expressions of uncertainty were coded to create a list of distinctly identifiable forms (concepts) of uncertainty. Because the data generated consisted of words rather than numbers, the findings are reported as descriptive summaries with general frequency patterns.

## Results

The findings on the theme of “uncertainty” fell under 3 subheadings: the world of uncertainty; how uncertainty impacted clients' feelings, thoughts, behaviors, attitudes, and personal expectations; and how uncertainty influenced clients' expectations of the oncology service. For reporting purposes, words in quotations are verbatim from the participants, and italics are used as a form of speech emphasis.

## The World of Uncertainty

### The Diagnosis of a Serious, Life-Limiting Cancer Shifted Participants Into a World of Uncertainty

Prior to the diagnosis, life in relation to their dog was lived following customary, taken-for-granted daily routines. For some, the clinical signs were imperceptible, with an incidental finding on examination or an acute hemorrhagic crisis as the first indication of threat. For others, the clinical signs were subtle, ill-defined, or vague, such as the gradual onset of lethargy or weight loss. For others yet, the clinical signs were assumed to be related to something “simple,” such as “an infection,” “arthritis,” or “constipation,” or the growth was assumed to be “innocent,” “just a tiny bump that had been there for months.” The diagnosis accordingly was nearly invariably experienced as “unexpected,” “traumatic,” and a “shock.” Participants described being “blown away” and “left reeling” by the news. One participant alleged the news of the diagnosis “hit” her “just as if it was a person.” In the midst of feeling “nervous,” “scared,” “tearful,” “overwhelmed,” and “devastated,” some posed the question, “Why me? Why us?” within a sense of existential “betrayal.” And with all participants equating cancer to a death sentence, the only certitude was the belief that their dog was “going to die,” and this was taken to be imminent. As one client recalled, “All I remember was crying because I thought [my dog] was going to die, really fast… My dog was dying and *that's all I knew*.”

### The Omnipresence of Uncertainty Was Strongly Evident in the Language of Participants

Along 3 broad subthemes, all the participants repeatedly stated, to various degrees and in relation to a range of specifics, that they “didn't know” (1) what was happening, (2) what to do, and (3) what to expect. *Not* knowing, participants were left “wondering,” “imagining,” “supposing,” and “guessing,” and lived in a world of “might(s),” “could(s),” “should(s),” “seems(s),” “maybe(s),” and “probably(s).”

Fifty-three content-distinct forms of uncertainty were identified (Table 1). Of these uncertainties, some would have been knowable and thus imminently addressed by the service (such as “What kind of cancer their dog had” and “What diagnostic tests might be needed”), others only knowable in a foreseeable future (such as “How well their dog would tolerate the chemotherapy” and “Whether the treatment protocol might need to be changed”), and the rest fundamentally unknowable (such as “What the cause of the cancer was” and “Whether diagnosing and/or treating the cancer earlier would have made a difference”). Note that the first 19 (36%) uncertainties relate to the general cancer experience, while the remaining 34 (64%) relate to engagement in the treatment of cancer.

**Table 1:** Fifty-three content-distinct forms of uncertainties expressed by 43 clients within 30 interviews comprising a qualitative study exploring client expectations at the oncology service of a tertiary referral center, the OVC Health Sciences Center, University of Guelph, Guelph, Ontario, April–October 2009^*^.

1. Whether a certain symptom was the cancer developing
2. Whether more overt symptoms would have led to an earlier diagnosis
3. *Whether the cancer was diagnosed at the earliest possible point in time*
4. Whether they responded to the cancer in a timely manner
5. *How long the cancer was there prior to diagnosis*
6. What the diagnosis would mean for their dog
7. What the diagnosis would mean for their family
8. *What was best and/or right for their dog*
9. What was best and/or right for the family
10. *How long their dog might live*
11. Whether their dog really had cancer
12. What kind of cancer their dog had
13. *What the cause of the cancer was*
14. What the natural biological behavior of the cancer was
15. How important certain symptoms of the cancer were to their dog's health and welfare
16. What stage the cancer was at
17. *Whether or when the cancer might metastasize or whether it has already metastasized*
18. *What the end would bring and how they would handle it*
19. *What the future would bring*
20. *What to expect as they access a specialty oncology service*
21. Who the staff were, what their positions were, and what responsibilities they held
22. When they see an oncologist versus a clinician versus a technician
23. When the behind-the-scenes contacts or activities were happening
24. How responsive and supportive the specialty service would be if or when called
25. What diagnostic tests might be needed
26. Which treatment option to choose
27. What treatment protocols were available
28. What the corresponding prognoses were
29. Whether they are making or made the best decisions
30. How concurrent conditions might influence the cancer, treatment plan or prognosis
31. Who they were really engaging in treatment for—themselves or their dog
32. What dogs on cancer treatment look like
33. *How well their dog would respond to treatment and achieve the desired efficacy and hoped for outcomes*
34. *How well their dog would tolerate the chemotherapeutic, radiation, surgical or palliative treatment*
35. *How to manage the day-to-day practicalities of living with a dog with cancer*
36. *What their dog's hospital and treatment experiences were like*
37. How their dog was feeling and what their dog was thinking
38. What long-term effects their dog might experience
39. What side-effects effects their dog might experience
40. How important certain side-effects were to their dog's health and welfare
41. If or when the side-effects would subside
42. Whether the medications to offset the side-effects would work
43. Why their dog was experiencing unexpected or unexplainable side-effects
44. How to differentiate whether subtle changes were cancer-related, treatment-related, or developmental
45. What the screening and monitoring tests might show
46. Whether the treatment protocol might need to be changed
47. *What or when the next steps in care would be*
48. *If or when the remission might break / (in the case of hemangiosarcoma) another bleed-out happen*
49. *When the time would arrive to stop treatment and/or euthanize*
50. Whether the service supported the end-of-life process (post-treatment)
51. Whether diagnosing and/or treating the cancer earlier would have made a difference
52. What the treatment ultimately would cost and what they would get from it
53. Whether, by a miracle or being ‘the exception,' the cancer might actually be cured

* *The italicized uncertainties were expressed by clients in all 3 stages (early, middle, and late) of the treatment program. These uncertainties may represent questions that are unanswerable (as in enduring uncertainties) or questions that need to be asked repeatedly throughout the cancer journey due to changing circumstances*.

### The Extent to Which the Participants Lived in Uncertainty Was Stage Dependent

The greatest degree of uncertainty expressed, as determined by examining the distribution of the uncertainties listed in Table 1, lay with those in the early stage, with 40 of 53 (75%) identified, followed by 27 of 53 (52%) and 36 of 53 (68%) in the middle and late stages, respectively.

## The Impact of Uncertainty on Feelings, Thoughts, Behaviors, Attitudes, and Personal Expectations

Living in a world of uncertainty strongly influenced participants' feelings, thoughts, behaviors, attitudes, and personal expectations, all of which impacted their lived experience, and thus, their ability to cope with, and adapt to, the realities of the cancer situation.

### Uncertainty Was Related to Feeling Anxious, Worried, and Fearful

In the participants' words, the feelings arising from their uncertainty included being “worried,” “scared,” “afraid,” “anxious,” and “concerned.” Two participants referred to “the fear of the unknown,” with one adding that “not knowing” was worse than knowing. The connection between “knowing” and “comfort” was present throughout the interviews, as evidenced in one client's comment, “The only way I'm going to get comfortable is [through] the more I know.” The discomfort with not knowing ranged from feeling mildly unsettled to highly distressed.

### Uncertainty Led to the Tendency to Catastrophize

Most participants were uninformed or misinformed about cancer and cancer treatment in dogs at the time of the diagnosis. Reflecting one extreme of naivety, one client, who described the diagnosis as “extremely unexpected” and “a shock,” explained, “We [the public] don't really think of dogs getting cancer.” Others assumed that cancer treatment in dogs would be “as taxing on the body” as it is with humans, and survival “tenuous at best.” As one client commented, “In fact, I suspect, sadly, it's interesting how it depends on what your information level is. Probably if we had known it was lymphoma, we would have let her die, because we wouldn't have known what we know now—the potential to give her chemo and it's not too toxic… and give her some reasonable lifespan. I would have thought, “Oh well, you know, I'm not going to torture her to keep her alive for 3 months.” Being uninformed or misinformed, the diagnosis was often concluded to be, as one client referred to it, “a big end-of-the-world thing.”

### Uncertainty Motivated Information Seeking in Multiple Directions, Including Resourcing the Primary Care Practitioner, Social Community, Internet, and Peers

#### The Primary Care Practitioner

Participants' first source of information was usually the primary care clinician. They spoke openly about the “bad news” consultation, for the most part satisfied with the information and guidance given.

##### The quality of the information and willingness to refer

Many participants deeply appreciated the option of referral to “the best place” for their dog's care. A few, however, expressed concerns about both the quality of the information given and their clinician's hesitancy to refer. There was evidence to suggest that a few referring veterinarians may have had inadequate knowledge about the specific cancer, its treatment, or both, were not aware of what speciality oncology services could offer, did not consider treatment or referral worthwhile, and were only referring out of pressure from their client. One client recalled her experience as follows:

“When {Patient} was first diagnosed, they weren't rushing us off to get chemotherapy treatment. So I'm not sure if every vet really makes the owners aware of what options are out there for their animals. Do they evaluate whether the couple would be willing to spend the money? I don't know what goes on in their decision-making, but I don't think the services are as well-known as perhaps they could be. They obviously knew that I wasn't willing to give up without giving her every possible chance. I'm wondering, why didn't they refer me to OVC? Why was it me having to keep fighting for her, pursuing it, that finally we came here?”

Another client shared how her practitioner's attitude changed. Pleased with the therapeutic outcomes, her practitioner confessed to her, “After seeing how well {Patient} has done, I would recommend cancer treatment at Guelph to everyone, whereas I wouldn't have done that before.”

##### Undue pessimism

Some participants were particularly distressed by what they thought were unduly pessimistic assessments by the primary care practitioner offering little to no hope, motivating 3 (10%) to seek a second opinion. One such participant, after a full year of remission and commitment to a second course of therapy (for lymphoma), recounted her experience, saying, “It was very upsetting for {the vet} to basically say that there was nothing we could do for {Patient}, that this is it and he's going to die essentially. Based on that experience…” she continued, “we ended up changing practices.” Another, after problem-free amputation and 6 months in remission (for osteosarcoma), shared her practitioner's original doubts, saying, “My vet did not think he'd be a good candidate for an amputation and it was just a matter of time, to wait, to see, to put him down. I didn't want to do that, so I came to Guelph… I came here a week later and they said, ‘Oh yes he would [be a good candidate].”' She then added, with intonation, “And you *might* base your judgement on what *certain people say*,” insinuating how clients might make treatment decisions based on their general practitioner's opinion, which, based on her experience, was not necessarily trustworthy, in that it lacked congruence with the opinion of the specialty service and the clinical outcomes achieved.

##### Client preparation for referral

The degree to which primary care practitioners informed and thus prepared participants for their referral visit to the oncology service ranged from a minimalist assurance to an accurate and precise account of what they might expect from the referral service. For one client, as recalled in his own words, “The vet just said, ‘You know what? Just go there. They know you're coming and they'll take care of you.' And that was all.” This is in stark contrast to another client's experience, as found in the following:

“She told me a lot of the things that they possibly would do and what to expect. I was prepared even before I came for hearing about the fact that they would do diagnostic testing and they would want to do ultrasound and they would need bloodwork and possibly more x-rays. So my home vet had prepared me for all [that]… So I came here knowing. And cost, too. She actually asked about those kinds of things. So my home vet actually laid a lot of the groundwork for what I was to find out here. So nothing was a surprise… She finds it important to know that you know what's going on, so she gives you lots of information. I think that's important, what you get at home to prepare you for coming here.”

The majority of participants had been, at most, minimally informed about what to expect at the oncology center. As a result, they had “no expectations” and “didn't know what to expect,” for as one client commented, “You can't have expectations unless you understand a bit about it.”

#### The Social Community

The search for information extended beyond the examination room, often in the form of conflicting, changeable, and exhaustive conversations with family members as they tried to make sense of the bad news. These exchanges opened up to extended family, friends, neighbors, and from there to “anyone” and “everyone” within the greater community, including co-workers, pet retail employees, groomers, people out on walks, and as one client said, “almost everybody that we could find that could relate to what we were going through.”

#### The Internet

With the desire to know as much as possible as quickly as possible, 15 (50%) participants had sourced the Internet. This client-driven independent research was powerful enough to not just gain an understanding of the cancer and its treatment options, but also directly influence the decision as to whether or not to pursue treatment. As one client admitted, “We originally didn't think that we were going to treat him, but then I did some research on-line…” Another client's research was so extensive that she had even chosen the specific protocol she wanted for her dog. In her words, “I spent ‘hours and hours'—‘days and days'—of research… so I was very well-researched before I came in… and I knew I wanted a certain protocol.”

Some participants' research was quite in-depth, involving looking up specific treatment protocols, side-effects and associated life expectancies. Other research centered on adjunctive support measures such as nutritional options (anti-oxidant diets, nutritional supplements), alternative approaches (naturopathy), and harm reduction methods (green disinfectants, natural candles, no perfumes). A few noted the risks with accessing web-based information, referencing the need to “read decent sites,” “validate the information,” and “take it with a grain of salt,” suggesting they were aware as opposed to naïve consumers.

#### Peers

It was clearly evident that all participants closely attended to others in the waiting room, zeroing in on the surrounding activities and one another's conversations, as well as engaging in mutual dialogue, all the while making internal comparisons in the effort to learn as much as possible. It was the participants with pets in the early stage of treatment who referred most frequently to their appreciation of information from others who were “in the same situation,” “boat” or “shoes,” especially if their peers were “a little further ahead.” The sharing and comparing of stories was variously described as “encouraging,” “beneficial,” and “reassuring.” It was a source of firsthand, tangible, experiential knowledge, as conveyed in one client's account, “You're just more informed about it, not from a doctor, but [from] somebody that's going through it.” As well, connecting with peers provided a sense of “camaraderie.” Through the similarities found within shared experience, the weight of isolation—of feeling singled out of the mainstream of the world of dog owners—lifted. As one client realized, “You're not alone… You know you're not the only one.”

### Uncertainty Motivated Vigilance

As was repeatedly evident in the participants' descriptions of their lifestyle and their perceptions of their dog's welfare, it was clear that they were closely monitoring their dogs. A number of clients felt that they had “missed” the presence of the cancer prior to the diagnosis, so they were especially vigilant after the diagnosis. What made the task challenging, compounding the uncertainty experienced, was that many patients exhibited minimal to no overt cancer-related illness or evidence of cancer, and tolerated treatment, such as chemotherapy, with little or only transitory treatment-related illness. They resumed “normal” lives and appeared disease-free, both physically and behaviorally. For many clients, the patients' cancer was imperceptible and thus difficult to gauge in the effort to maintain an accurate perspective of the situation. As one client commented, “She looks so wonderful, you would never dream she has this cancer in her body… It's almost like a fake world that we're living in.”

Thus, even with close vigilance, many participants found themselves perplexed. The lack of clear-cut, concrete, observable evidence made it difficult for them to truly gauge their dog's cancer status or monitor disease progression. It made it difficult for them to distinguish between the clinical signs of cancer, side-effects of treatment, and other conditions, such as the onset of an illness or a natural age-related developmental change such as arthritis, lower energy or increased sleeping, especially when the signs were so subtle they could suggest any number of possibilities. Within subtlety, some clients even began to question normal behaviors, second-guessing the range of what was previously considered normal for their dog.

### Uncertainty Led to Various Degrees of Denial, Avoidance, Selective Ignoring, Selective Misinterpretation, or Minimization

Just a few participants demonstrated overt evidence of these emotion-focused coping strategies, based on analysis of single interviews and not purposely screening for this during the interviews. Note that clients' distinct efforts to avoid specific aspects of their informational realities did not necessarily preclude specific information-seeking activities, such as the continuous monitoring of their dog's quality of life (especially since this was the foremost qualification in engaging in and continuing care). The two approaches to coping were not mutually exclusive.

For one client, the invisibility of the cancer permitted her and her husband to “pretend” that their dog was “not sick.” When asked how she managed to live with a dog with an incurable cancer, she answered, through tears, “The answer's funny. You pretend he's not sick, because hemangiosarcoma is the type of cancer where, when he's not doing chemo, you can pretend he's not sick, cause you don't see it and it doesn't make him sick… We pretend, and we do it every day… You live in denial, but prepare for the worst.” Pretending provided these clients temporary relief from the anticipatory grief they were experiencing.

For others, the invisibility risked the erroneous conclusion that the situation was stable, even when they had been advised otherwise. As one client commented, having been forewarned that another episode of abdominal hemorrhage could occur at any time, “I guess I sort of buried that somewhere… I might have been a little in denial… I just kept thinking it was going to be somehow farther away. And when that [bleed] happened, it was just like a slap, a big slap—wake-up call.”

In these cases, the first client expressed conscious awareness of the strategy used, while the second appeared unaware, leading him to be caught off guard when reality caught up. In this instance, despite his use of the term “denial,” it is impossible to say, based on an excerpt alone, whether he had truly “denied” the information given, or had engaged in avoidance, selective ignoring or misinterpretation, or minimization. Any of these strategies could equally have been in play.

### Uncertainty Gave Rise to Hopeful, Optimistic Attitudes

Without certitude upon which to rely, some participants appeared to intentionally choose an optimistic “way of thinking.” This was seen as necessary, as found in, “We have just a total positive outlook on this. You need that.” Others felt the same, saying, “You have to think positive” and “You've got to be optimistic.” Some chose optimism to add force to the fight. As one said, “The mind is a very powerful weapon against diseases or anything you're up against—you can be a negative or a positive person… I think I'm pretty positive.” Some clients who noticed the usual, happy-go-lucky way their dog continued on with life—unaffected by the diagnosis—intentionally adjusted their own perspective, taking on a mutual collaboration to go on with life “as per usual,” not letting the reality of cancer interfere with living life in a full, happy “Carpe diem—Seize the day!” kind of way.

### Uncertainty Led to Wishful Thinking

Some participants toyed with thoughts of cure, thinking that *their* dog, out of all dogs, might be “the exception.” Eight (27%) of the participants (3 early, 2 middle, and 3 late stage) expressed purported beliefs that their dog could be “the exception to the rules,” “the one that will beat the odds,” and “the one-in-a-million to be cured.” Three (10%) clients went so far as to say they were “hoping for a miracle.”

### Uncertainty Telescoped Life Into the Present

With a future filled with uncertainty, described as, “a big question mark,” all the participants reined in their worlds to the present, adjusting their expectations of their dog and their life with their dog. Since the unpredictability of the future negated any perceived hold on it, they relinquished it to instead plant their expectations into the envisionable present. As one client said, “It is just unpredictable, so we just do it day-to-day and see how he does.” Others echoed this, referring to living “day-to-day,” “week-to-week,” and “from one treatment to another.” There were no “expectations” or “plans” for the future. As one client said, “I try not to look too far in the future. You don't know what's around the corner.”

### Uncertainty Appraisals: The Meaning Attributed to Uncertainty

For the greatest part, the participants of the current study had appraised the uncertainty as a danger. This was evident with the fear, distress, and tendency to catastrophize at the time of diagnosis, as well as the multiple coping strategies drawn on, consciously or unconsciously, in their efforts to manage the uncertainty and adjust to the situation. These included the problem-focused strategies of seeking information, accessing social support; closely monitoring their dogs; intentionally adopting an optimistic attitude; and living in the present. They also included the emotion-focused strategies of denial, avoidance, selective ignoring or misinterpretation, and minimization, as well as wishful thinking. It is important to recognize that optimism may equally have originated from the appraisal of uncertainty as an opportunity rather than danger. Without certainty to delimit outcomes, uncertainty may have been envisioned as a well of hope for all that might be possible, recognizing that the limits of individuals can surpass the confinements of statistics.

## The Influence of Uncertainty on Client Expectations of the Oncology Service

Since living in a world of uncertainty strongly informed participants' feelings, thoughts, behaviors, attitudes, and personal expectations, it powerfully influenced their expectations of the oncology service.

### Participants Wanted Information About All Aspects of the Cancer Journey

Most participants wanted as much information as possible about the following: (1) the cancer and natural course of events without treatment, (2) treatment options and associated prognoses, including the pragmatics of financial, time, and emotional investment, (3) diagnostics and monitoring, potential side-effects and complications, expected time-lines for treatment response, remission and relapse, and potential for cure, (4) parameters for monitoring and measuring quality of life, and, (5) when “enough is enough,” when it would be “right” and “best” to discontinue treatment and/or euthanize.

### Participants Specifically Wanted to Know About Their Dog's Hospital Experience

Some participants expressed concern about not knowing what happened behind closed doors. The participants' experience was that dogs *go*—are taken away from them for treatment—and *come back*—are returned to them to go home. Depending on mind-set, the space in between could be filled with many imaginings. Some clients wondered “where” their dog was and “how” the treatment was administered, as found in one client's admission, “I do wonder what it's like and how they do it… So I'm like, ‘Where's he sitting?' like, ‘Where is he?' What I imagine is that they have little cubicles with IV bottles and whatever in each, and there's a wall, but they can maybe see each other and hear each other. Like, just cubicles. And there's all these cubicles—that's what I imagine.”

Time in hospital was time beyond the seeable, hearable, and touchable, and thus knowable. One client worded this quite poignantly in the following commentary:

“They come and take your dog away and you're already heartbroken… They said we couldn't go in… It would have been nice if we could watch some of the procedure through a glass window or something… even if we just saw the first procedure so we know what was happening so we feel a little more comfortable… I'm *not* happy with not having *any* contact at *all*… because, our time with her is *limited* now, for sure, and I would like to know what's happening *to* her, making sure that I'm *comfortable* with what I'm doing to her… I've made the step to try and prolong her life, but I want to make sure that I'm not sacrificing the *quality* of life… I know you can't have people in the way when you're trying to work. I understand that. There are times when it won't work, but I'm sure that the professionals can figure out which times don't work. It can't be *all* the time. I think there should be some flexibility, especially when you know the pet is terminal.”

This client strongly felt services should “try to maintain the bond” as much as possible, rather than take your dog away “to places” “for procedures” of which they had little understanding.

### Participants Specifically Wanted Information About the Potential Treatment Outcomes

For each participant, being informed about the potential treatment outcomes, most particularly the side-effects, meant being prepared. One client described it as having “ammunition” or “strategies,” saying, “{Technician} anticipates problems in a way that's very helpful and gives you ammunition or strategies to help you deal with these problems before they happen and you have no idea what to do.” Another said, “And they were very good about laying that out: ‘You may experience this. You may experience that. If you experience this, you need to do *A*. If you experience that, you need to do *B*.”' Being fully prepared meant being prepared “for the worst.” Most participants did not want, as one client called it, “the ‘middle of the line' stories.” They felt better knowing—and thus being prepared for—the worst that could happen. As one client shared, “I need to know the worst-case scenario… If I can prepare for it, but hope for the best, then I feel more comfortable.”

### Participants Specifically Wanted to Know About the Transition From Active Treatment Aimed to Extend Life to Palliative or End-of-Life Care

For many, since the non-curative nature of the cancer remained a non-dismissible reality, they questioned what “the inevitable end” might bring and how they would handle it. Hand-in-hand with the decision to start treatment was the need to know when to end it, and the imperative, thus, to be informed about quality of life and the transition to palliative or end-of-life care, as well as be assured of the continuing support of the oncology team. Although the forward thrust of exam room conversations was on the preservation of life, with the collaborative goal to extend quantity of quality life, there appeared to be an equal need, at least for some, to openly address the transition to end-of-life. As one client said, “Not that we wanted to hear it, but we needed to know.”

### Participants Wanted, Needed, and Appreciated Service With Those They Knew and Trusted

For each and every participant, relationship was a source of comfort and confidence, and the foundation for trust in the service. As one client shared, “They know who I am, and there's just that awareness now… [We] know each other and know what to expect.” This client thought it would be “very hard” on somebody to have to “adjust” and form “a new bond” partway through with new service providers. For one client who did need to form “a new bond,” she repeatedly described how “apprehensive” she was. She questioned the new technician's competency, how well she would get to know her dog, and how prompt she would be with home support, reflecting the instant introduction of layers of uncertainty and a breach of confidence while the rest of the service remained constant.

### Participants Needed and Depended Upon the Continuous Access to Informational Support in Case of an Unexpected Event or Complication

Participants who engaged in the earnest endeavor of chemotherapy, radiotherapy, or major surgery needed to know they could access “advice,” “direction,” and “guidance” should they find themselves worried about their dog's condition or an adverse event or crisis develop. With cancer behavior and treatment response incompletely predictable, despite standardized, proven treatment protocols, every participant needed ongoing access to informational support in order to manage the eventualities that might arise. One client likened it to “a crutch,” saying, “Even if you don't use it, it's still there.” The ability to lean on the service in time of need was imperative to the sense of security needed to engage in the cancer treatment journey.

### Participants Wanted and Appreciated Timely Information and Service

Waiting represented time in uncertainty, time spent “in the unknown.” The undercurrent of anxiety that characterized the uncertainty of the cancer context intensified when the participants were kept waiting. They most especially spoke to the “stress of waiting” in reference to incidents when the news to be received was potentially bad, as with diagnostic and screening tests. Waiting for return phone calls when “in a crisis” at home was also cited as stressful. As one participant observed, “Waiting for a phone call back seems like hours… Every second seems like hours.”

## Discussion

For the participants of this study, the diagnosis of a life-limiting cancer in a much-loved dog represented a crisis in their lives, abruptly shifting them from a world characterized by continuity, orderliness, and coherence to a world set apart by ambiguity, unpredictability, and ominous probabilities (6, 51). With cancer metaphorically associated with death (52), the only certainty was death.

The omnipresence of uncertainty was evident in the choice and frequency of the terminology used and the content discussed. Uncertainties were expressed in relation to the cancer and its treatment, especially the latter, suggesting the extent to which engagement in a treatment program co-engages an entirely new dimension of unknowns, magnifying the degree of, and complexity of, uncertainties experienced. While the most uncertainty was experienced in the early stage of treatment, the level rose again at the late stage to approximate the level originally experienced. Evidence for this same pattern of uncertainty is found in the human medical literature (9, 17). In a qualitative study by Cohen (17) of the parental uncertainty experienced with children having life-limiting childhood illness, uncertainty was experienced “most acutely and unrelentingly” when the disease was newly diagnosed (17). It dissipated to lower, more constant levels over time with increasing familiarity with the disease. It could, however, be easily triggered by exacerbations of the disease, relapses, and the beginning of downhill trajectories, as well as the conclusion of the treatment program (that had effected a beneficial response) and the associated regularly scheduled diagnostic tests (that provided unambiguous evidence of health). Our findings appear to parallel those of Cohen (17), with the initial uncertainty dissipating to relatively lower, stable levels with increasing familiarity with the diagnosis, treatment program, and cancer service; and then rising with the impending discontinuation of the treatment program and diagnostic monitoring, and anticipation of disease exacerbation or relapse and the eventual transition to end-of-life care, especially given the limited prognoses of these patients.

Mishel's *Uncertainty in Illness Theory* (9, 10) proposes uncertainty as the central psychological feature of the illness experience (11), needing to be effectively managed in order to successfully adapt to the illness experience. Indications of difficulty with adaptation are not in relation to the uncertainty itself, but to how well it is managed (10, 11). Consequently, the veterinary profession's awareness of its role, responsibility, and potential to support client efforts to manage uncertainty is of paramount significance to the welfare of clients.

Services unaware of clients' struggles with uncertainty or insensitive to the challenges therein may inadvertently contribute to the burden of uncertainty, and undermine, as opposed to support client welfare. Fundamental to this end, oncology services can be designed to support client needs to reduce uncertainty when appraised as a danger, and sustain or increase uncertainty when appraised as an opportunity. In this way services can facilitate *adaptive uncertainty management* (18, 53, 54). Each of these approaches will be discussed in turn.

### Uncertainty Reduction: Information Management

As noted in earlier publications (42, 43) and likewise reported by Neville (29), with the goal of supporting client efforts to reduce uncertainty, it is important to start by determining clients' needs for information, information preferences, and perceptions of uncertainty. This can be accomplished by first exploring the client's perspective of the cancer situation with open-ended questions (55), and listening for what they know, as well as don't know and would like to know. For example, “*What are you hoping to learn in this appointment?” “What do you know about this cancer*?” “*What did your referring veterinarian tell you?”* and “*How has learning about your dog's cancer impacted you?”* Next, a statement acknowledging how some clients “like to know a lot,” while others “prefer just the basics” can be made, and their individual preference sought (55). A clinician could enquire, “*What would you prefer — the big picture or all the details?”* Lastly, offering a statement of normalization, acknowledging the reality that in cancer care (as with life in general) not all answers are always possible, would be appropriate. For example, “*We know much more today, however there is still a lot that we do not know.”* This could be followed by, “*How do you find the ‘not knowing' part of things?”* inviting their perspective and listening for the degree to which this may be difficult for them and how they typically manage such circumstances (55). Since perceptions of illness-related uncertainty may vary (19), some clients may want uncertainty reduced as much as possible while others may see benefit in it. Such awareness can be used to guide information giving, keeping in mind the balance between clients' need to know and their fear of knowing, as mentioned in Stoewen et al. (42, 43) and reported by Kodish and Post (56). Just as information, for some, can be empowering, for others, it can be incapacitating (6, 18). This could be explored, for example, by offering, “*For some clients, information can be empowering, and for others it can be overwhelming. Where do you stand?”*

Unfortunately, a simple direct correlation between “information giving” and “uncertainty reduction” does not exist. Providers of information need to be aware of the potential limitations in uncertainty reduction (19, 57). For instance, as described in previous reports (42, 43) and elsewhere (19, 57), too much information can confuse and overwhelm, leading to information overload, diminishing the ability to critically assess, organize and prioritize information into an understandable format. Likewise, the complexity of the information given can give rise to new, unanticipated uncertainties, and the reduction of one uncertainty can potentially lead to a cascade of subsequent uncertainties (19). As can be appreciated, uncertainty reduction is not necessarily a straightforward process. Ironically, information can not only reduce, but also increase uncertainty.

To minimize this potential, attention to the quality and structure of the information is necessary. Quality relates to the “sufficiency” (clarity, completeness and volume) and the “reliability and validity” (accuracy, source ethos, ambiguity, applicability, and consistency) of the information (57). Structure relates to the order in which the particulars are presented (57). The provision of clear, accurate, complete, consistent, and appropriately measured increments of applicable information, structured in a logical format, can go far to support uncertainty reduction. As mentioned in Stoewen et al. (42, 43), this must occur within the boundaries of clients' information preferences. Information given in a staged approach called “chunking and checking,” (55) alternating between providing small amounts of information and checking for understanding, will ensure the client's views and preferences are kept central to the information-giving process, maximizing the potential to meet information preferences. To incorporate the client's interpretation and interests to direct the information-giving process, a clinician could periodically ask, “*What questions do you have?” “What additional information may I provide?”* or “*What needs further clarification?”* using client response to guide the amount and type of information provided and avoid inadvertently compounding the uncertainty experienced.

#### Providing Information to Ensure Informed Decisions

Information giving serves to ensure clients are able to make fully informed decisions (42, 43). The decision of greatest consequence occurs at the time of diagnosis with the choice of the degree and type of intervention. With clients potentially uninformed or misinformed about cancer in dogs and equating the diagnosis to a death sentence and treatment to suffering, the tendency to catastrophize can negate the option to treat, risking patient welfare and premature termination of the human-animal bond. Thus, the imperative, whether at the primary care or specialty setting, is to provide the best possible client education.

Verbal information supported with visuals, such as diagrams and jointly reviewed articles, and supplemented with take-home written materials and recommendations to informative websites are recommended (58). Maintaining availability via offering or planning a follow-up phone conversation or appointment past the breaking-the-bad-news event permits clients' questions or concerns to be addressed (58). Clients want, need and have the right to accurate, objective, up-to-date information with supportive guidance to help them weigh the risks and benefits of the various options for care (42, 43). The veterinary-client exchange at this critical juncture is of profound importance in determining the short- and long-term consequences for the health and welfare of the patient and the patient's family.

#### Providing Information to Prepare Clients

Information giving also serves to ensure clients will be prepared for the outcomes of those decisions. Preparatory information reduces uncertainty by providing an orientation to the future, and in so doing, increases familiarity with what the future might bring (9, 41). According to the findings of the current study, and supported by the literature (16, 41), if the decision is made to refer to a specialty service, such orientation can and should begin at the primary care service. The provision of an accurate and precise account of what to expect at the specialty center will familiarize clients with this new experience. Orientation efforts should then continue at the referral service, enabling clients to navigate a healthcare system much broader than what they are familiar with (16, 59). Helpful options could include a new client information package and/or admission orientation program.

The current study suggests that clients may want to be informed about the *entire* cancer treatment journey: the in-hospital treatment process, the potential outcomes and how they should be managed, and the transition from active treatment to palliation or euthanasia. Oncology services can do clients a great service by helping them understand their dog's hospital experience. Without adequate knowledge, clients wonder and even worry about where their dog is, what is happening, and how their dog is coping, all uncertainties that are reducible and are even preventable with a measure of client education. Educational efforts necessarily must start with sufficient explanations, but could also include supplementary resources such as pamphlets or booklets, videos of the hospital and treatment programs, a walk-through hospital tour, and an informative website with a video or virtual tour (60, 61). In addition to educational efforts, hospital service can be designed in a client-friendly manner to preserve the bond. This could include bond-responsive measures such as hospital design that features observation windows and hospital policies that minimize separation time and maximize client engagement in patient care.

Educating clients about the potential outcomes of the treatment program, both effects and side-effects, enables them to be prepared for all eventualities: good, bad, and inconsequential. This can help ready them for the challenges that may lie ahead (42, 43). Perhaps less acknowledged is the importance of preparing clients for the results of diagnostic screening tests and any potential resultant adjustments to treatment. Prior to tests being run, clients should be reminded about the range of results possible and what they would mean to avoid unnecessary additional uncertainty and distress when the news received is unfavorable (62). For clients wanting to be fully prepared, the full spectrum of possibilities—including the worst possible case scenarios—should be discussed. According to the results of this study, the English proverb, “Hope for the best, but prepare for the worst,” seems material in the veterinary oncology context.

Since clients with dogs with incurable cancer maintain a long-range perspective on the cancer situation, it seems imperative for oncology services to follow this lead in order to complement and address client concerns. Making space for conversation about the full journey of care right from the beginning will give clients the permission to raise their concerns as they arise, reducing the burden of uncertainties and supporting their efforts to prepare for the future.

#### Supporting Client Information Seeking

As well as reducing uncertainty through the provision of information, oncology services can intentionally support clients' own efforts to reduce uncertainty in two important ways. First, with the Internet as a readily available, expedient, and powerfully influential source of information, services can relegate their role as *providers* to instead be interpreters and integrators of information (63, 64). Services can empower clients through acknowledging their proactive efforts, facilitating the interpretation and integration of information, and directing them to recognized, high-quality official websites (63). Note that since the dawn of the Internet there has been increasing adoption. At the time of data collection for this study (2009), just 50% of the participants had accessed the Internet for information. Today it appears that the vast majority of clients access the Internet for information (65), making the shift from providers to interpreters and integrators of information all that much more important.

And second, with clients wanting to connect with peers for information and social support, options such as offering peer group meetings, a buddy support system, a web-based chat site, or a cozy, relaxing café-style waiting area for clients whose dogs are day-hospitalized may support clients' efforts to reduce uncertainty. Connection permits social comparison, which can reduce uncertainty (9, 41, 66), but it can also positively influence appraisals of, and responses to, uncertainty (67, 68), and in this way advantageously contribute to adaptation on multiple levels. All information seeking and information sharing efforts should be validated, encouraged, and facilitated.

### Uncertainty Reduction: Service Provision

#### Ensuring Continuing Relationships

Integral to services with the aim to reduce uncertainty is the imperative to provide clients with an oncology care team that remains consistent throughout the provision of service. The relationship with the service provider is the foremost means to prevent uncertainty (41), making this vital to services committed to uncertainty reduction. Continuing relationships not only reduce relational uncertainty (69, 70), but also build trust and confidence in service providers, which reduces the overall burden of uncertainty (68).

#### Providing Ongoing Informational Support

Also, integral in the aim to reduce uncertainty is the provision of 24-h informational support. This is not only of importance in the case of true emergencies, but also in situations of more benign consequence that are plagued by uncertainty. The combination of fear, vigilance, and perplexity makes it easy for clients to interpret changes in their dog as more severe or serious than they really are. Because emotional arousal and selective attention can cause perceptual and cognitive biases (9), clients need the continuity of service to assist them with interpreting their observations, and through such, alleviate uncertainty.

#### Minimizing Waiting

Lastly, attention to the timeliness of service is integral in the aim to reduce uncertainty. Waiting, which is recognized as a problematic phenomenon in the medical healthcare fields (71), tends to share three common features: uncertainty, threat, and powerlessness, which in combination result in psychological distress (72). Service designed with an emphasis on continuity along all aspects of service time lines—including, for example, the time between determining the need for a test, performing the test, and providing the results—can minimize wait times, and in so doing, prevent unnecessary uncertainty.

### Uncertainty Maintenance and Promotion

Uncertainty reduction is not always possible, both in relation to the nature of illness uncertainty and the limitations of information giving. Clients must live with and manage a significant burden of uncertainty along the cancer journey, which, when appraised as a danger, can significantly compromise quality of life (16, 73). Uncertainty, however, by its very nature, is open to multiple interpretations (10). Likewise, these interpretations are not fixed, but can change, both over time (10, 74) and as a consequence of social interaction (18, 54).

#### Supporting a Positive Orientation to Uncertainty

Recognizing the plasticity of appraisals makes real the potential to improve client quality of life through supporting positive and thus more adaptive appraisals of uncertainty when uncertainty is irreducible. Service providers should intentionally look for appropriate opportunities to partake in maintaining and even promoting uncertainty by adopting and encouraging a positive orientation toward it (10, 16, 73).

#### Supporting Client Efforts to Cope

Moreover, uncertainty reduction is not always in the client's best interests, particularly in situations of an unwelcomed, negative certainty, or when uncertainty is appraised as an opportunity (9). In such situations, in order to forestall the perception of the unwelcomed certainty and facilitate hope, clients may attempt to regulate information using emotion-focused coping strategies such as denial, avoidance, selective ignoring, minimizing, and selective misinterpretation (9). Repeated efforts on the part of well-intentioned service providers to reduce uncertainty could undermine clients' best efforts to cope. In this case, rather than overwhelm clients with unwanted details and destroy hope, uncertainty could, to some degree, intentionally be preserved by providing no more than the essential facts, thus supporting, rather than undermining, efforts to cope (19). Furthermore, assisting clients to identify and engage in alternative, perhaps more adaptive, coping strategies may better enable effective adaptation over the long run.

## Conclusion

Veterinary oncology clients need to adapt to the cancer experience. Health care service providers can enhance or hinder the adaptation process. In providing services, it is important for service providers to focus on the *relational* aspects of care—“the covenant between care giver and care receiver”—as well as the *transactional* aspects of care—the set of “efficient auditable transactions between consumers and providers” (75). Understanding clients' experience of illness uncertainty—what it is and how it manifests (information seeking, optimism, vigilance, denial, selective ignoring, minimizing, and wishful thinking)—can not only enable adaptive uncertainty management, but engender a more patient, understanding and compassionate approach to the provision of services.

Over three decades have passed since Madewell stated in his commentary, “Though the *science* of veterinary oncology is new to clinical practice, the *psychology* of veterinary oncology is virtually unexplored” (76). With the identification of uncertainty as the overarching psychological feature of the veterinary client experience in the specialty oncology setting, we believe the current study marks a turning point. Through rigorous analytic techniques, we were able to generate an in-depth understanding of a previously unreported, and in the authors' experience under-recognized, aspect of client experience that profoundly impacts their world, who they are, and what they bring to and expect from the oncology service. Through identifying the psychological phenomenon of uncertainty, we have attained a new understanding of the subjective experience of clients, and through such, the evidence upon which to design and deliver services.

The limitations of this study should be considered. The findings of qualitative research, which focuses on specific individuals within specific contexts, cannot be easily generalized to other individuals or contexts. Generalizability of qualitative research depends on the extent to which the research context and the specific context, wherein the findings may be applied, are similar (44). Care thus must be taken to consider the applicability of our findings prior to extrapolating them to other cancer care situations (44).

The small number of participants in the present study, and the fact that they were mostly female and all very attached to their dogs, may not necessarily represent the overall population of clients seeking cancer care services for dogs at tertiary referral centers. Caution is always required when generalizing findings derived from one clinical setting to another (77, 78).

Although researcher bias always exists, to reduce the potential, a prior literature review was purposefully avoided. Instead, the present study was approached with a de novo (i.e., naïve) mindset. The interviews were deliberately carried out in a client-driven manner, enabling significant client control of the direction of the interview and content discussed. In addition to this, an intentionally curious, dispassionate, and non-partisan stance was maintained. Of importance, because the purpose of the study was to evaluate the expectations of clients, not the experience of uncertainty, the interview process and data analysis were not intended to identify uncertainty. The concept of uncertainty arose emergently as a thematic finding.

Further research is needed to explore the concept of illness uncertainty in veterinary medicine. There is no reason to believe that clients within other health care settings, such as emergency or primary care practice, or clients with pets that have illnesses other than cancer, do not experience illness uncertainty or would not benefit from communication practices that could help them manage uncertainty. Research in other settings and contexts in veterinary medicine would be beneficial. Research into the impact of gender and attachment on uncertainty would also be beneficial.

To further the understanding of illness uncertainty in veterinary oncology, a qualitative or mixed methods approach to specifically study clients' self-reported experience of uncertainty would enable a deeper understanding of the nature and sources of uncertainty, variations with stage of illness (e.g., diagnosis, treatment, and posttreatment), appraisals of uncertainties, coping strategies used, and preferences for communication support. Such research could have important implications for theories of health communication.

There is a “crucial connection” between uncertainty and communication (79), that, when understood, can inform the strategies upon which to facilitate the management of uncertainty in ways that will enhance clients' psychological well-being, and enable the opportunity to support clients' successful adaptation to the cancer experience. Research in human medicine has aimed not only to understand illness uncertainty, but also to assess interventions (80, 81) and offer evidence-based recommendations for the provision of service which may alleviate its impact (16, 17). Since communication is integral to the construction, management, and resolution of uncertainty (11), research has specifically branched to explore the communication strategies that may support the ability to manage it (11, 19, 57, 82).

Given the increasing attachment of people to their pets (83, 84), the increasing prevalence of cancer in the pet population (84, 85), and the ever-increasing options for cancer treatment, the demand for cancer care will continue to rise, further challenging the profession to attend to both the science *and* the psychology of veterinary oncology. Research in the area of veterinary psycho-oncology may be in its beginnings; however, the potential to advance the evidence upon which best practices can be built, in order to truly deliver on the promise of bond-centered care, lies at hand.

## Data Availability

The datasets for this study will not be made publicly available because the authors do not have participant consent to provide these. Participant consent extends to verbatim quotes only. The provision of entire transcripts would risk the chance of participant identification.

## Author Contributions

DS is the corresponding author and did the research and writing of the article as part of a Ph.D. thesis. JC and CD were DS' graduate co-supervisors and were involved extensively throughout all phases of the research and writing of this article. CM and ES were graduate committee members and provided feedback in developing the research proposal, critiqued aspects of data analysis, and supported revisions of the paper. All authors have given approval for the article submitted for publication.

### Conflict of Interest Statement

The authors declare that the research was conducted in the absence of any commercial or financial relationships that could be construed as a potential conflict of interest.

## Abbreviations

OVC: Ontario Veterinary College
LAPS: Lexington Attachment to Pets Scale.
